# Supplementary material for: Insights into Genetic and Antigenic Characteristics of Influenza A(H1N1)pdm09 Viruses Circulating in Sicily During the Surveillance Season 2023–2024: The Potential Effect on the Seasonal Vaccine Effectiveness
Source: Viruses. 2024 Oct 21;16(10):1644. doi: 10.3390/v16101644 (PMC11512306; doi:10.3390/v16101644)
Supplement: Supplementary file 1 [file viruses-16-01644-s001.zip › viruses-3232709-supplementary.pdf]

Table S1. Primers and probes used for RNAse P, detection and subtyping of influenza A and influenza B.

RNase P

Primer	Sequence (5' to 3')
RNase P For	AGA TTT GGA CCT GCG AGC G
RNase P Rev	GAG CGG CTG TCT CCA CAA GT
RNase P Probe	Cy5 – TTC TGA CCT GAA GGC TCT GCG CG – BHQ-1

Influenza A and B detection

Primer	Sequence (5' to 3')
InfA For1 Primer	CAA GAC CAA TCY TGT CAC CTC TGA C
InfA For2 Primer	CAA GAC CAA TYC TGT CAC CTY TGA C
InfA Rev1 Primer	GCA TTY TGG ACA AAV CGT CTA CG
InfA Rev2 Primer	GCA TTT TGG ATA AAG CGT CTA CG
InfA Probe	FAM –TGC AGT CCT CGC TCA CTG GGC ACG – BHQ-1
InfB For Primer	TCC TCA AYT CAC TCT TCG AGC G
InfB Rev Primer	CGG TGC TCT TGA CCA AAT TGG
InfB Probe	JOE – CCA ATT CGA GCA GCT GAA ACT GCG GTG – BHQ-1

Influenza A subtyping

Primer	Sequence (5' to 3')
A(H1N1)pdm09	
H1pdm For Primer	GTG CTA TAA ACA CCA GYC TCC CAT T
H1pdm Rev Primer	AGA YGG GAC ATT CCT CAA TCC TG
H1pdm Probe	FAM – TGG CCA GYC "T" CA ATT TTG TGC TTT TTA CAT A *
A(H3N2)	
H3 For Primer	AAG CAT TCC YAA TGA CAA ACC
H3 Rev Primer	ATT GCR CCR AAT ATG CCT CTA GT
H3 Probe	FAM – CAG GAT CAC A"T"A TGG GSC CTG TCC CAG *

* "T" residue modified with an internal Black Hole Quencher-1 (10th nucleotide) and with a Spacer C3 at the 3'-end

Influenza B subtyping (lineage)

Primer	Sequence (5′ to 3′)
Victoria lineage	
B/VIC For Primer	GAT CTG GAT GTA GCC TTG GG
B/VIC Rev Primer	TAA CAG GTC TGA CTT CAT GGA G
B/VIC Probe	FAM – TTC CCT GTR CAT TTT G – BHQ-1
Yamagata lineage	
B/YAM For Primer	GAT CTG GAT GTG GCC TTG GG
B/YAM Rev Primer	AGG TCT GAC YTC GTG RAG TA
B/YAM Probe	FAM – CAC ACA CAT TGG CCT – BHQ-1

Table S2. Protocol used for multi-segment reverse transcription-PCR (M-RTPCR) of influenza A viruses.

Primer	Sequence (5′ to 3′)
Uni12/Inf-1	GGG GGG AGC AAA AGC AGG
Uni12/Inf-3	GGG GGG AGC GAA AGC AGG
Uni13/Inf-1	CGG GTT ATT AGT AGA AAC AAG G

These primers target the conserved regions of 12 nucleotides at the 3′ terminus and 13 nucleotides at the 5′ of each of the eight segments of influenza A genome.

In short, each M-RTPCR reaction was performed using the SuperScript™ IV One-Step RT-PCR System (Invitrogen, Waltham, MA, USA) in a final volume of 25 µL, including 12,5 µL of Platinum SuperFi RT-PCR Master Mix, 0,5 µL of SuperScript IV RT Mix, 0,2 µL of 10 µM Uni12/Inf-1, 0,3 µL of 10 µM Uni12/Inf-3, 1 µL of 10 µM Uni13/Inf-1, 5 µL of RNA template and 6,5 µL of nuclease-free water.

The thermal profile was as follows: 42°C for 60 min, 94°C for 2 min, and then 5 cycles of 94°C for 30 s, 44 °C for 30 s and 68 °C for 3 min, followed by 35 cycles of 94°C for 30 s, 55°C for 30 s, 68°C for 3 min, with a final extension at 68°C for 5 min.

Table S3. Patient's info and GISAID accession numbers of eight segments of influenza A(H1N1)pdm09 viruses collected in Sicily during the season 2023-2024

Patient's info		Influenza A(H1N1)pdm09									
**Age (years)**	**Vaccinated**	**Strain name**	**GISAID ID**	**PB2**	**PB1**	**PA**	**HA**	**NP**	**NA**	**MP**	**NS**
53	No	A/Agrigento/105690/2023	EPI_ISL_19327639	EPI3495214	EPI3495215	EPI3495216	EPI3495218	EPI3495217	EPI3495219	EPI3495220	EPI3495221
9	No	A/Agrigento/106188/2023	EPI_ISL_19327662	EPI3495222	---	EPI3495223	EPI3495224	EPI3495225	EPI3495226	EPI3495227	EPI3495228
5	No	A/Agrigento/106189/2023	EPI_ISL_19327706	EPI3495229	---	EPI3495230	EPI3495231	EPI3495232	EPI3495233	EPI3495234	EPI3495235
92	No	A/Agrigento/106419/2023	EPI_ISL_19327752	EPI3495236	EPI3495237	EPI3495238	EPI3495239	EPI3495240	EPI3495241	EPI3495242	EPI3495243
96	Yes	A/Catania/106036/2023	EPI_ISL_19327877	EPI3495244	---	EPI3495245	EPI3495246	EPI3495247	EPI3495248	EPI3495249	EPI3495250
7	No	A/Catania/106129/2023	EPI_ISL_19327878	EPI3495251	---	---	EPI3495252	EPI3495253	EPI3495254	EPI3495255	EPI3495256
4	No	A/Catania/106699/2023	EPI_ISL_19327879	EPI3501476	---	EPI3501477	EPI3501478	EPI3501479	EPI3501480	EPI3501481	EPI3501482
9	Yes	A/Catania/106704/2023	EPI_ISL_19327880	EPI3501483	---	EPI3501484	EPI3501485	EPI3501486	EPI3501487	EPI3501488	EPI3501489
12	No	A/Catania/106779/2023	EPI_ISL_19327881	EPI3501490	EPI3501491	EPI3501492	EPI3501493	EPI3501494	EPI3501495	EPI3501496	EPI3501497
83	No	A/Catania/106783/2023	EPI_ISL_19327882	EPI3501498	EPI3501499	EPI3501500	EPI3501501	EPI3501502	EPI3501503	EPI3501504	EPI3501505
1	No	A/Catania/107539/2024	EPI_ISL_19327883	EPI3501506	EPI3501507	EPI3501508	---	EPI3501509	---	EPI3501510	EPI3501511
71	Yes	A/Catania/107853/2024	EPI_ISL_19327884	EPI3501512	---	EPI3501513	EPI3501514	EPI3501515	EPI3501516	EPI3501517	EPI3501518
64	Yes	A/Enna/105929/2023	EPI_ISL_19327885	EPI3501519	---	EPI3501520	EPI3501521	EPI3501522	EPI3501523	EPI3501524	EPI3501525
53	Yes	A/Enna/106169/2023	EPI_ISL_19327886	---	---	EPI3501526	EPI3501527	EPI3501528	EPI3501529	EPI3501530	EPI3501531
17	No	A/Messina/106230/2023	EPI_ISL_19327888	EPI3501532	---	EPI3501533	EPI3501534	EPI3501535	EPI3501536	EPI3501537	EPI3501538
2 months	No	A/Messina/106520/2023	EPI_ISL_19327889	---	---	---	---	---	---	EPI3501539	---
1	No	A/Messina/107233/2024	EPI_ISL_19327890	EPI3501540	---	---	EPI3501541	EPI3501542	EPI3501543	EPI3501544	EPI3501545
1	No	A/Messina/107873/2024	EPI_ISL_19327892	---	---	---	EPI3501546			EPI3501547	EPI3501548
6	No	A/Palermo/105654/2023	EPI_ISL_19327895	EPI3501572	---	EPI3501574	EPI3501575	EPI3501576	EPI3501577	EPI3501578	EPI3501579
8 months	No	A/Palermo/105656/2023	EPI_ISL_19327896	EPI3501580	---	EPI3501581	EPI3501582	EPI3501583	EPI3501585	EPI3501589	EPI3501591
3	No	A/Palermo/105910/2023	EPI_ISL_19327897	---	---	---	EPI3501595	---	---	EPI3501596	EPI3501597
40	No	A/Palermo/106054/2023	EPI_ISL_19327898	EPI3501599	---	EPI3501600	EPI3501601	EPI3501602	EPI3501603	EPI3501604	EPI3501605
11	No	A/Palermo/106080/2023	EPI_ISL_19327899	EPI3501606	---	EPI3501607	EPI3501609	EPI3501611	EPI3501613	EPI3501615	EPI3501617
19	No	A/Palermo/106152/2023	EPI_ISL_19327900	EPI3501628	---	---	---	---	EPI3501631	EPI3501632	EPI3501633
12	No	A/Palermo/106200/2023	EPI_ISL_19327901	EPI3501634	---	EPI3501635	EPI3501636	EPI3501637	EPI3501638	EPI3501639	EPI3501640
3	No	A/Palermo/106208/2023	EPI_ISL_19327902	EPI3501641	---	EPI3501642	EPI3501643	EPI3501644	EPI3501645	EPI3501646	EPI3501647
10 months	No	A/Palermo/106209/2023	EPI_ISL_19327903	EPI3501648	---	EPI3501649	EPI3501650	EPI3501651	EPI3501652	EPI3501653	EPI3501654
3	No	A/Palermo/106213/2023	EPI_ISL_19327904	EPI3501655	---	EPI3501656	EPI3501658	EPI3501660	EPI3501663	EPI3501665	EPI3501667
2	No	A/Palermo/106286/2023	EPI_ISL_19327905	EPI3501670	---	EPI3501671	EPI3501672	EPI3501673	EPI3501674	EPI3501675	EPI3501676

Patient’s info		Influenza A(H1N1)pdm09									
**Age (years)**	**Vaccinated**	**Strain name**	**GISAID ID**	**PB2**	**PB1**	**PA**	**HA**	**NP**	**NA**	**MP**	**NS**
4	No	A/Palermo/106288/2023	EPI_ISL_19327906	EPI3501682	---	EPI3501684	EPI3501686	EPI3501688	EPI3501689	EPI3501690	EPI3501691
7	No	A/Palermo/106289/2023	EPI_ISL_19328003	EPI3501692	---	EPI3501693	EPI3501694	EPI3501695	EPI3501696	EPI3501697	EPI3501698
2	No	A/Palermo/106432/2023	EPI_ISL_19328004	EPI3501701	---	EPI3501702	EPI3501703	EPI3501704	EPI3501705	EPI3501706	EPI3501707
2	No	A/Palermo/106472/2023	EPI_ISL_19328005	EPI3501708	---	EPI3501709	EPI3501710	EPI3501711	EPI3501712	EPI3501713	EPI3501714
8	No	A/Palermo/106495/2023	EPI_ISL_19328006	EPI3501715	---	EPI3501716	EPI3501717	EPI3501718	EPI3501719	EPI3501720	EPI3501721
2	No	A/Palermo/106507/2023	EPI_ISL_19328007	---	---	EPI3501722	EPI3501723	---	---	EPI3501724	EPI3501725
1	No	A/Palermo/106511/2023	EPI_ISL_19328008	---	---	------	---	---	---	EPI3501726	---
7	No	A/Palermo/106513/2023	EPI_ISL_19328009	EPI3501727	---	EPI3501728	EPI3501729	EPI3501730	EPI3501731	EPI3501732	EPI3501733
9	No	A/Palermo/106514/2023	EPI_ISL_19328010	EPI3501734	---	EPI3501735	EPI3501736	EPI3501737	EPI3501738	EPI3501739	EPI3501740
7 months	No	A/Palermo/106594/2024	EPI_ISL_19328011	EPI3501741	---	EPI3501742	EPI3501743	EPI3501744	EPI3501745	EPI3501746	EPI3501747
3	No	A/Palermo/106598/2024	EPI_ISL_19328028	EPI3501748	---	EPI3501749	EPI3501750	EPI3501751	EPI3501752	EPI3501753	EPI3501754
5	No	A/Palermo/106634/2023	EPI_ISL_19328042	EPI3501755	EPI3501756	EPI3501757	EPI3501758	EPI3501759	EPI3501760	EPI3501761	EPI3501762
5	No	A/Palermo/106637/2023	EPI_ISL_19328054	EPI3501763	---	---	EPI3501764	EPI3501765	EPI3501766	EPI3501767	EPI3501768
6	No	A/Palermo/106644/2023	EPI_ISL_19328063	EPI3501769	---	EPI3501770	EPI3501771	EPI3501772	EPI3501773	EPI3501774	EPI3501775
9	No	A/Palermo/106673/2024	EPI_ISL_19328073	EPI3501776	---	EPI3501777	EPI3501778	EPI3501779	EPI3501780	EPI3501781	EPI3501782
3	No	A/Palermo/106681/2024	EPI_ISL_19328082	---	---	EPI3501783	EPI3501784	EPI3501785	EPI3501786	EPI3501787	EPI3501788
2	No	A/Palermo/106693/2024	EPI_ISL_19328092	EPI3501789	---	EPI3501790	EPI3501791	EPI3501792	EPI3501793	EPI3501794	EPI3501795
1	Yes	A/Palermo/106742/2024	EPI_ISL_19328101	---	---	EPI3501796	EPI3501797	EPI3501798	EPI3501799	EPI3501800	EPI3501801
6 months	No	A/Palermo/106763/2024	EPI_ISL_19328112	EPI3501802	EPI3501803	EPI3501804	EPI3501805	EPI3501806	EPI3501807	EPI3501808	EPI3501809
4	No	A/Palermo/106766/2024	EPI_ISL_19328123	EPI3501810	EPI3501811	EPI3501812	EPI3501813	EPI3501814	EPI3501815	EPI3501816	EPI3501817
79	No	A/Palermo/106942/2024	EPI_ISL_19328133	EPI3501818	EPI3501819	EPI3501820	EPI3501821	EPI3501822	EPI3501823	EPI3501824	EPI3501825
7	Yes	A/Palermo/107123/2024	EPI_ISL_19328149	EPI3501826	EPI3501827	EPI3501828	EPI3501829	EPI3501830	EPI3501831	EPI3501832	EPI3501833
1	No	A/Palermo/107124/2024	EPI_ISL_19328160	EPI3501834	---	EPI3501835	EPI3501836	EPI3501837	EPI3501838	EPI3501839	EPI3501840
9	No	A/Palermo/107126/2024	EPI_ISL_19330419	EPI3501841	---	EPI3501842	EPI3501843	EPI3501844	EPI3501845	EPI3501846	EPI3501847
8	No	A/Palermo/107218/2024	EPI_ISL_19330426	EPI3501848	EPI3501849	EPI3501850	EPI3501851	EPI3501852	EPI3501853	EPI3501854	EPI3501855
7	No	A/Palermo/107282/2024	EPI_ISL_19330441	EPI3501856	---	EPI3501857	EPI3501858	EPI3501859	EPI3501860	EPI3501861	EPI3501862
2	No	A/Palermo/107283/2024	EPI_ISL_19330442	EPI3501863	EPI3501864	EPI3501865	EPI3501866	EPI3501867	EPI3501868	EPI3501869	EPI3501870
2 months	No	A/Palermo/107286/2024	EPI_ISL_19330443	EPI3501871	EPI3501872	EPI3501873	EPI3501874	EPI3501875	EPI3501876	EPI3501877	EPI3501878
3	No	A/Palermo/107287/2024	EPI_ISL_19330452	EPI3501879	EPI3501880	EPI3501881	EPI3501882	EPI3501883	EPI3501884	EPI3501885	EPI3501886
9 months	No	A/Palermo/107332/2024	EPI_ISL_19330463	EPI3501887	EPI3501888	EPI3501889	EPI3501890	EPI3501891	EPI3501892	EPI3501893	EPI3501894

Patient’s info		Influenza A(H1N1)pdm09									
**Age (years)**	**Vaccinated**	**Strain name**	**GISAID ID**	**PB2**	**PB1**	**PA**	**HA**	**NP**	**NA**	**MP**	**NS**
11	No	A/Palermo/107405/2024	EPI_ISL_19330473	EPI3501895	---	EPI3501896	EPI3501897	EPI3501898	---	EPI3501899	EPI3501900
1	No	A/Palermo/107541/2024	EPI_ISL_19330483	---	---	---	EPI3501901	---	EPI3501902	EPI3501903	EPI3501904
9	No	A/Palermo/107571/2024	EPI_ISL_19330493	EPI3501905	---	EPI3501906	EPI3501907	EPI3501908	EPI3501909	EPI3501910	EPI3501911
10	No	A/Palermo/107573/2024	EPI_ISL_19330504	EPI3501912	EPI3501913	EPI3501914	EPI3501915	EPI3501916	EPI3501917	EPI3501918	EPI3501919
10 months	No	A/Palermo/107898/2024	EPI_ISL_19330528	EPI3501920	EPI3501921	EPI3501922	EPI3501923	EPI3501924	EPI3501925	EPI3501926	EPI3501927
4	No	A/Palermo/108000/2024	EPI_ISL_19330539	EPI3501928	---	EPI3501929	EPI3501930	EPI3501931	EPI3501932	EPI3501933	EPI3501934
7	No	A/Palermo/108070/2024	EPI_ISL_19330549	EPI3501935	EPI3501936	EPI3501937	EPI3501938	EPI3501939	EPI3501940	EPI3501941	EPI3501942
62	No	A/Siracusa/107428/2024	EPI_ISL_19330560	EPI3501943	---	EPI3501944	EPI3501945	EPI3501946	EPI3501947	EPI3501948	EPI3501949
12	No	A/Siracusa/107876/2024	EPI_ISL_19330571	EPI3501950	EPI3501951	EPI3501952	EPI3501953	EPI3501954	EPI3501955	EPI3501956	EPI3501957
15	No	A/Siracusa/108015/2024	EPI_ISL_19330580	EPI3501958	---	EPI3501959	EPI3501960	EPI3501961	EPI3501962	EPI3501963	EPI3501964
14	No	A/Trapani/105912/2023	EPI_ISL_19330591	EPI3501965	---	EPI3501966	EPI3501967	EPI3501968	EPI3501969	EPI3501970	EPI3501971
6	No	A/Trapani/106245/2023	EPI_ISL_19330602	EPI3501972	---	EPI3501973	EPI3501974	EPI3501975	EPI3501976	EPI3501977	EPI3501978
2	No	A/Trapani/106607/2024	EPI_ISL_19330611	EPI3501979	---	---	EPI3501980	EPI3501981	EPI3501982	EPI3501983	EPI3501984
4	No	A/Trapani/106612/2024	EPI_ISL_19330621	EPI3501985	---	---	EPI3501986	EPI3501987	EPI3501988	EPI3501989	EPI3501990
9 months	No	A/Trapani/106631/2023	EPI_ISL_19330634	EPI3501991	---	EPI3501992	EPI3501993	EPI3501994	EPI3501995	EPI3501996	EPI3501997
1	No	A/Trapani/107169/2024	EPI_ISL_19330643	EPI3501998	---	EPI3501999	EPI3502000	EPI3502001	EPI3502002	EPI3502003	EPI3502004
1	No	A/Trapani/107205/2024	EPI_ISL_19330653	EPI3502005	---	EPI3502006	EPI3502007	EPI3502008	---	EPI3502009	EPI3502010
33	No	A/Trapani/108213/2024	EPI_ISL_19330667	---	---	---	---	---	---	EPI3502011	EPI3502012

Abbreviations: PB2, polymerase basic 2; PB1, polymerase basic 1; PA, polymerase acid; HA, hemagglutinin; NP, nucleocapsid protein; NA, neuraminidase; MP, matrix protein; NS, nonstructural protein

Table S4. Frequency of AA substitutions identified in A(H1N1)pdm09 strains from Sicily (other than HA and NA segments) during the season 2023-2024 (only mutations with frequencies ≥10% are reported).

Segment	Amino acid substitution	Frequency (%)
**1 – PB2, polymerase basic 2**	I81M/T	64.2
	R251K	16.4
	L475M	46.3
	T662A/S	17.9
**2 – PB1, polymerase basic 1**	K57T	65.0
	N213D	15.0
	K757R	60.0
**3 – PA, polymerase acid**	V14I	27.7
	M61I	44.6
	L63I	43.1
	K213R	38.5
	R262K	44.6
	K339N	29.2
	V407I	15.4
	L482M	40.0
**5 – NP, nucleoprotein**	E14D	46.4
	T22A	46.4
	I33V	40.6
	G34S	15.9
	I136L	46.4
	V217I	46.4
	Q453P	91.3
**8 – NS, non-structural protein**	W102C (NS1)	16.0
	Q109K (NS1)	37.3
	S212P (NS1)	82.7
	G67E/K (NS2/NEP)	84.0
	Q85H (NS2/NEP)	45.3
	I106L (NS2/NEP)	45.3
